# Transcriptomic and proteomic analyses of ovarian follicles reveal the role of VLDLR in chicken follicle selection

**DOI:** 10.1186/s12864-020-06855-w

**Published:** 2020-07-16

**Authors:** Qiuyue Chen, Yiya Wang, Zemin Liu, Xiaoli Guo, Yi Sun, Li Kang, Yunliang Jiang

**Affiliations:** 1 Shandong Provincial Key Laboratory of Animal Biotechnology and Disease Control and Prevention, College of Animal Science and Veterinary Medicine, Shandong Agricultural University, Taian, China; 2 College of Life Science, Qi Lu Normal University, Jinan, China

**Keywords:** Chicken, Follicle, Proteome, Transcriptome, Differentially expressed genes, Differentially expressed proteins

## Abstract

**Background:**

Follicle selection in chickens refers to the process of selecting one follicle from a group of small yellow follicles (SY, 6–8 mm in diameter) for development into 12–15 mm hierarchical follicles (usually F6 follicles), which is an important process affecting laying performance in the poultry industry. Although transcriptomic analysis of chicken ovarian follicles has been reported, integrated analysis of chicken follicles for selection by using both transcriptomic and proteomic approaches is still rarely performed. In this study, we compared the proteomes and transcriptomes of SY and F6 follicles in laying hens and identified several genes involved in chicken follicle selection.

**Results:**

Transcriptomic analysis revealed 855 differentially expressed genes (DEGs) between SY follicles and F6 follicles in laying hens, among which 202 were upregulated and 653 were downregulated. Proteomic analysis revealed 259 differentially expressed proteins (DEPs), including 175 upregulated and 84 downregulated proteins. Among the identified DEGs and DEPs, changes in the expression of seven genes, including *VLDLR1, WIF1, NGFR, AMH, BMP15, GDF6* and *MMP13*, and nine proteins, including VLDLR, VTG1, VTG3, PSCA, APOB, APOV1, F10, ZP2 and ZP3L2, were validated. Further analysis indicated that the mRNA level of chicken VLDLR was higher in F6 follicles than in SY follicles and was also higher in granulosa cells (GCs) than in thecal cells (TCs), and it was stimulated by FSH in GCs.

**Conclusions:**

By comparing the proteomes and transcriptomes of SY and F6 follicles in laying hens, we identified several differentially expressed proteins/genes that might play certain roles in chicken follicle selection. These data may contribute to the identification of functional genes and proteins involved in chicken follicle selection.

## Background

The ovary is a dynamic organ and a pivotal component of the reproductive system in hens. In the abdomen of laying hens, ovarian follicles of various sizes exist, including small white follicles that are less than 3 mm in diameter, large white follicles that are 3–5 mm in diameter, small yellow (SY) follicles that are 6–8 mm in diameter, large yellow follicles that are 9–12 mm in diameter and five to six hierarchical follicles of increased sizes, i.e., F6 to F1 [1]. Follicle selection in reproductively active domestic hens refers to the daily collection of one follicle from a pool of 6–8 mm small yellow follicles, which becomes a hierarchical follicle [2] and continues to develop rapidly from the F6 follicle stage to the F1 follicle stage until ovulation. In the process of chicken follicle selection, granulosa cells rapidly proliferate and differentiate to produce high levels of progesterone [3, 4], and vitellin synthesized by the liver enters oocytes via the very low-density lipoprotein receptor (VLDLR) [5, 6].

Changes in the transcripts involved in steroidogenesis, paracrine signaling and transcription during the early stage of follicular growth and development were identified by transcriptome analysis [7]. Comparison among the transcriptomes of small white, F1 and postovulatory chicken follicles identified differentially expressed genes that are involved in the adhesion, apoptosis and steroid biosynthesis pathways [8]. Candidate genes, including ANXA2, Wnt4 and transforming growth factor genes, were shown to play several roles in chicken follicle growth [9–14]. However, the dynamics of the transcriptome during chicken follicle development from the SY follicle to the F6 follicle are unclear.

In addition, the mRNA abundance may not accurately predict the quantities of the corresponding functional proteins, while a proteomic approach can provide a systemic overview of protein levels [15]; therefore, a proteomic approach has certain advantages over mRNA expression profiling [16]. Proteomic analyses of ovarian function [17] and maturation of oocytes [18], such as polycystic ovarian syndrome (PCOS) and cancer [19], in human ovarian diseases and early embryonic development [20–22] in mammals were reported, while in chicken, 2889 proteins were identified in the white yolk and ovarian stroma of small white follicles in Bovan’s white laying hen [23]. However, the temporal changes in the proteome during chicken follicle selection are unknown. In this study, we compared the proteomes and transcriptomes of 6–8 mm SY follicles and the smallest hierarchical follicles (F6) in laying hens and found several differentially expressed genes/proteins (DEGs/DEPs) that might play certain roles in chicken follicle selection.

## Results

### Transcriptomic analysis

RNA-seq was used to compare the transcriptomes of three SY follicles and three of the smallest hierarchical follicles (F6), which are referred to here as S1, S2, and S3 and F1, F2 and F3, respectively. High-throughput RNA-seq generated 61.66 Gb of clean data from the six samples of chicken follicles, and 91.07–93.42% of the reads could be mapped to the chicken genome. For all six samples, at least 93.55% of the reads were equal to or exceeded Q30 (Table 1).

**Table 1:** Summary of the RNA-seq metrics for chicken follicles

Sample	Total reads	Mapped reads, %	Unique mapped reads, %	GC content, %	% ≥ Q30
S1	62,822,554	57,509,858 (91.54%)	56,245,873 (89.53%)	50.83	93.75
S2	65,638,382	59,775,235 (91.07%)	58,415,734 (89.0%)	51.07	93.55
S3	67,279,424	61,497,217 (91.41%)	60,160,054 (89.42%)	50.39	93.55
F1	66,848,634	61,330,120 (91.74%)	59,870,822 (89.56%)	50.93	93.71
F2	67,659,778	62,284,292 (92.06%)	60,929,361 (90.05%)	50.65	94.01
F3	80,778,754	75,462,807 (93.42%)	73,776,873 (91.33%)	50.06	94.24

**Fig. 1:**
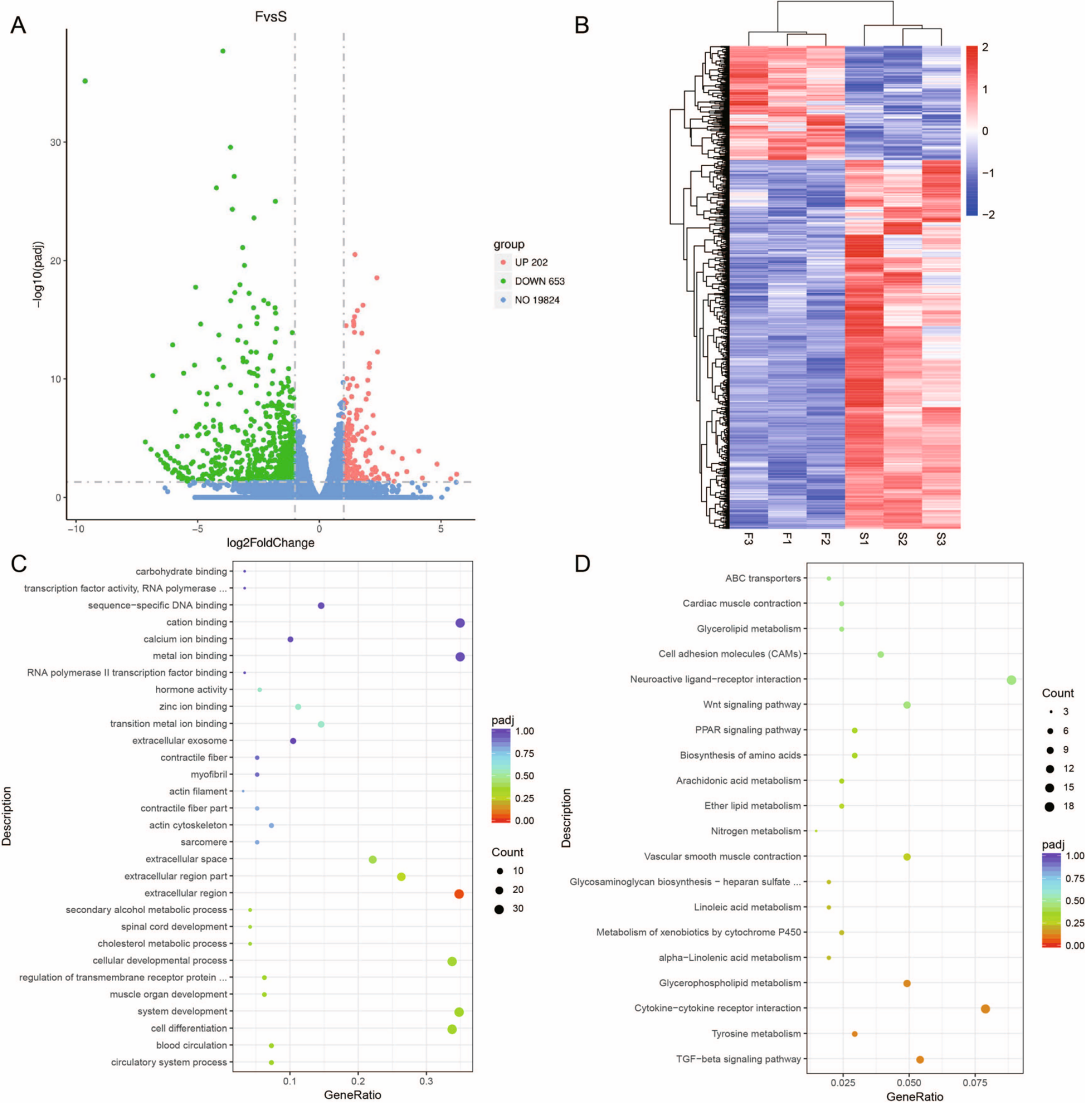
Transcriptome profile comparison between SY follicles (S) and F6 follicles (F). **a** Volcano plot of all the genes detected in six chicken follicle samples. Green spots represent downregulation, and red spots represent upregulation. **b** Hierarchical clustering analysis of DEGs between F and S. **c** GO enrichment of DEGs from the F and S transcriptomes. **d** KEGG signaling pathway enrichment analysis of DEGs

A total of 855 DEGs, including 202 upregulated and 653 downregulated genes, were identified between the SY follicles (S) and F6 follicles (F) according to the significance criteria of |log_2_ (FoldChange)| > 1 and padj < 0.05 (Fig. 1a). A hierarchical clustered map of DEGs was then constructed and is shown in Fig. 1b. Detailed analysis of the top 10 up−/downregulated DEGs is shown in Table 2. The entire list of DEGs is shown in Table S1.

**Table 2:** The top 10 up- and downregulated genes of chicken F6 vs SY follicles

Gene name	Gene ID	log_2_ FoldChange	*P*-value	padj	Regulated
*KRT75L2*	431,299	5.6448359195	0.00089581216788	0.010968512776	Up
*SBK2*	420,929	5.4012005063	0.0028916599926	0.026749987217	Up
*TYRP1*	395,913	4.0884568588	3.25E-06	0.00011942416133	Up
*INHA*	424,197	3.5955900397	0.0004632196608	0.0065938632923	Up
*ACTC1*	423,298	3.4047368046	1.87E-05	0.0005136082463	Up
*SPTSSB*	425,008	3.1879775126	0.00086973054149	0.010723329659	Up
*DMRT2*	100,858,556	3.0375474936	4.20E-06	0.00014842983586	Up
*EGR4*	422,950	2.6567614753	0.00082143038769	0.01027699966	Up
*MFSD2B*	428,656	2.5659125526	1.55E-06	6.55E-05	Up
*GJD2*	395,273	2.4535441052	0.00290562839884	0.0268098383411422	Up
*SPIRE1L*	418,362	−7.1514399248	4.01E-07	2.06E-05	Down
*SOX3*	374,019	−6.9225703884	2.13E-06	8.52E-05	Down
*MLPH*	424,019	−6.3375597357	4.03E-06	0.00014300529036	Down
*POU4F3*	395,521	−6.319423575	7.90E-05	0.0016173871827	Down
*LHX3*	373,940	−6.2949552193	0.00012211061251	0.0022934186943	Down
*HAUS3L*	101,751,348	−6.1283949694	1.72E-05	0.00048016708266	Down
*GCNT3*	427,492	−6.0294054072	3.27E-16	1.36E-13	Down
*GNOT2*	396,117	−5.9594390066	3.33E-05	0.00082793115753	Down
*CDH15*	107,054,331	−5.8252534515	1.13E-06	5.03E-05	Down
*EIF4E1B*	107,054,521	−5.7904110653	5.48E-05	0.0012171943551	Down

The DEGs were then assessed by GO and KEGG pathway analyses. The GO functional analysis revealed that most of the DEGs were involved in circulatory system processes, cell differentiation and transition metal ion binding (Fig. 1c). KEGG pathway analysis of the DEGs showed that the most enriched pathways were those involved in TGF-β signaling, tyrosine metabolism and cytokine-cytokine receptor interactions (Fig. 1d).

**Fig. 2:**
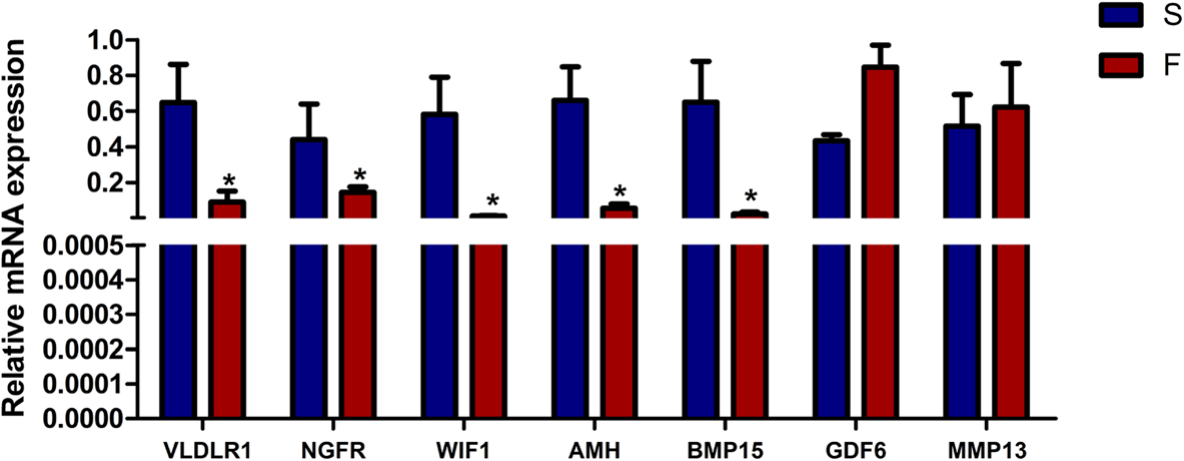
The mRNA expression levels of genes examined by qRT-PCR. All data are presented as the mean ± SEM. *, *P* < 0.05

To validate the RNA-seq data, seven DEGs (Table S2), including very low density lipoprotein receptor 1 (*VLDLR1*), nerve growth factor receptor (*NGFR*), WNT inhibitory factor 1 (*WIF1*), anti-Mullerian hormone (*AMH*), bone morphogenetic protein 15 (*BMP15*), growth differentiation factor 6 (*GDF6*) and matrix metallopeptidase 13 (*MMP13*), were chosen and quantified by quantitative real-time PCR (qRT-PCR). The results showed that the mRNA levels of these genes were similar to those revealed by the sequencing data, suggesting that the RNA-seq results were reliable (Fig. 2).

### Proteomics analysis

**Fig. 3:**
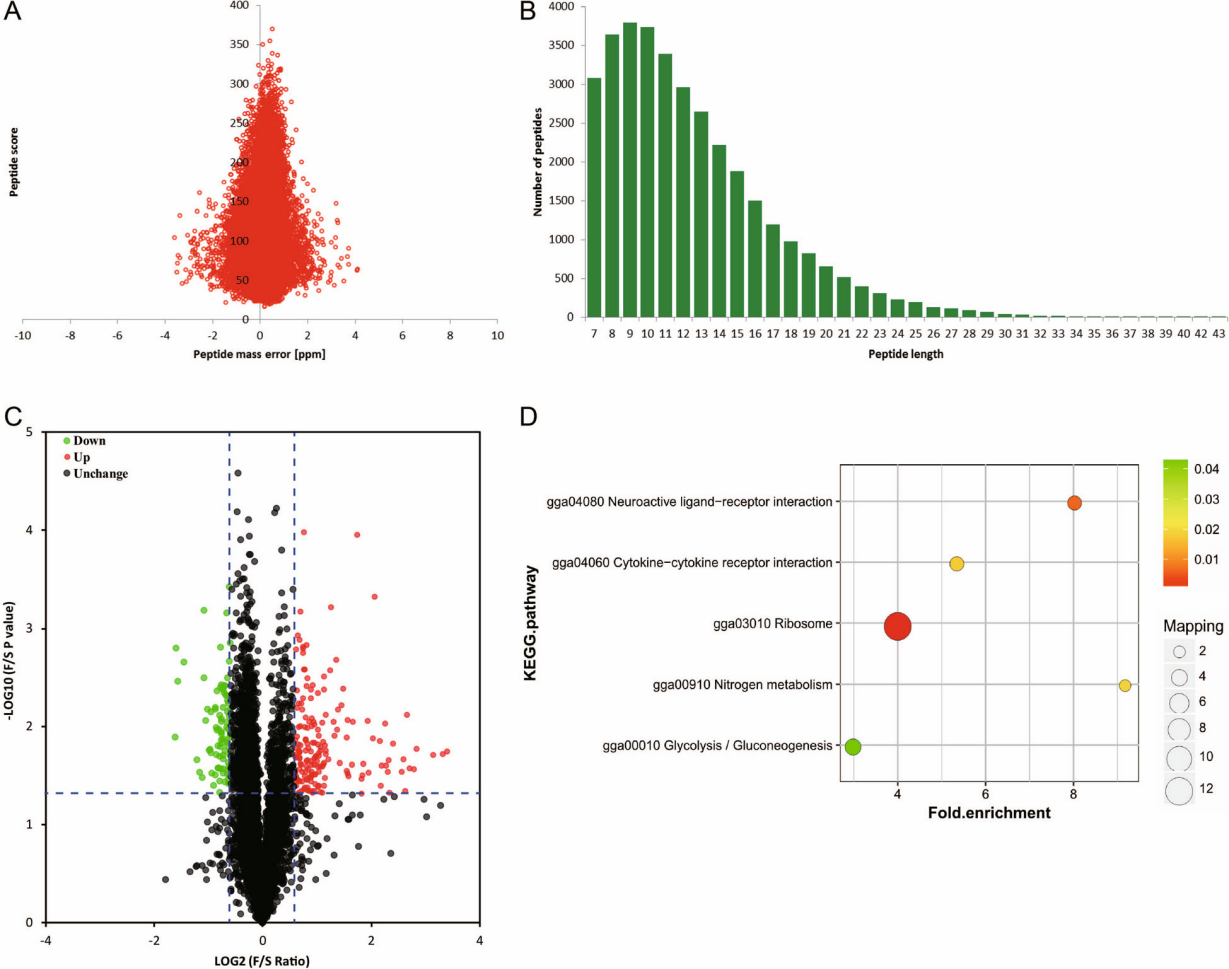
TMT analysis of the DEP data for the chicken F6 and SY follicles. **a** Mass error distribution of all identified peptides. **b** The length distribution of the majority of the peptides. **c** Volcano plots of -log_10_ (*P* value) versus log_2_ (expression level) in the F6 vs SY follicles. **d** KEGG signal pathway enrichment analysis of the DEPs

The proteins from three SY follicles and three of the smallest hierarchical follicles (F6) that were used for the above transcriptome analysis, i.e., the S1, S2, and S3 and F1, F2 and F3 follicles, were used for TMT labeling and HPLC fractionation followed by LC-MS/MS analysis. The first step was to validate the MS data. The distribution of the mass error was close to zero, and most of the absolute values were less than 5 ppm, which meant that the mass accuracy of the MS data was compliant with the requirements (Fig. 3a). The length of most peptides was between eight and 16 amino acids, which was in agreement with the general characteristics of tryptic peptides (Fig. 3b). In this study, a total of 5883 proteins were identified in the samples, and 5236 proteins were quantified. According to the relative levels, the quantified proteins were divided into two categories: proteins with a quantitative ratio over 1.5 were considered upregulated, and proteins with a quantitative ratio less than 1/1.5 were considered downregulated (*P* < 0.05) (Fig. 3c). In the F6 follicles, the levels of 175 and 84 proteins were significantly increased and decreased, respectively, compared with those in the SY follicles. A detailed analysis of the top 10 up−/downregulated differentially expressed proteins (DEPs) is shown in Table 3. The entire list of DEPs is shown in Table S3.

**Table 3:** Top 10 up- and downregulated proteins in chicken F6 vs SY follicles

Protein accession code	Protein description	Protein name	F/S ratio	F/S *P* value	Regulation
P02659	Apovitellenin-1	–	10.52430044	0.018057763	Up
A0A1D5NZ61	Kinesin-like protein KIF20B	–	9.951417004	0.019122892	Up
A0A1D5PYJ4	Transcriptional repressor p66-alpha	GATAD2A	8.772228989	0.019458417	Up
P41366	Vitelline membrane outer layer protein 1	VMO1	7.163371488	0.016976433	Up
F1NV02	Apolipoprotein B	APOB	6.922300706	0.026918903	Up
R4GM71	Phosphatidylcholine-sterol acyltransferase	LCAT	6.531100478	0.026742079	Up
A0A1L1RYU0	Prostate stem cell antigen	PSCA	6.297115385	0.007551582	Up
A0A1D5NVU2	Keratin, type II cytoskeletal 75	KRT75	6.183636364	0.045556059	Up
P25155	Coagulation factor X	F10	6.062727273	0.021845706	Up
A0M8U1	Suppressor of tumorigenicity 7 protein homolog	ST7	5.905829596	0.02867129	Up
A0A1L1RJJ7	Wnt inhibitory factor 1	WIF1	0.327939317	0.012827638	Down
A0A1D5P589	Tudor and KH domain-containing protein	TDRKH	0.331994981	0.001609664	Down
A0A1D5P0E3	Epithelial cell adhesion molecule	EPCAM	0.367965368	0.00222955	Down
A0A1D5PS81	Protein LSM14 homolog B	LSM14B	0.43363064	0.02217556	Down
F1NWH5	Aquaporin-3	AQP3	0.447158789	0.02917623	Down
F1NC54	SH3 domain and tetratricopeptide repeat-containing protein 1	SH3TC1	0.462576038	0.03313208	Down
E1C8L9	Vacuolar protein sorting-associated protein 29	VPS29L	0.475690608	0.003217184	Down
C7ACT2	Unknown	LOC422926	0.475784992	0.000664487	Down
F1N9X0	Folate receptor alpha	FOLR1	0.482795699	0.008705237	Down
F1P337	Sorting nexin	SNX5	0.49547821	0.00675724	Down

The pathways of the DEPs were constructed using KEGG software. Several important pathways were enriched in the F6 follicles compared with the SY follicles (Fig. 3d), including pathways involved in the ribosome, neuroactive ligand-receptor interactions and cytokine-cytokine receptor interactions.

**Fig. 4:**
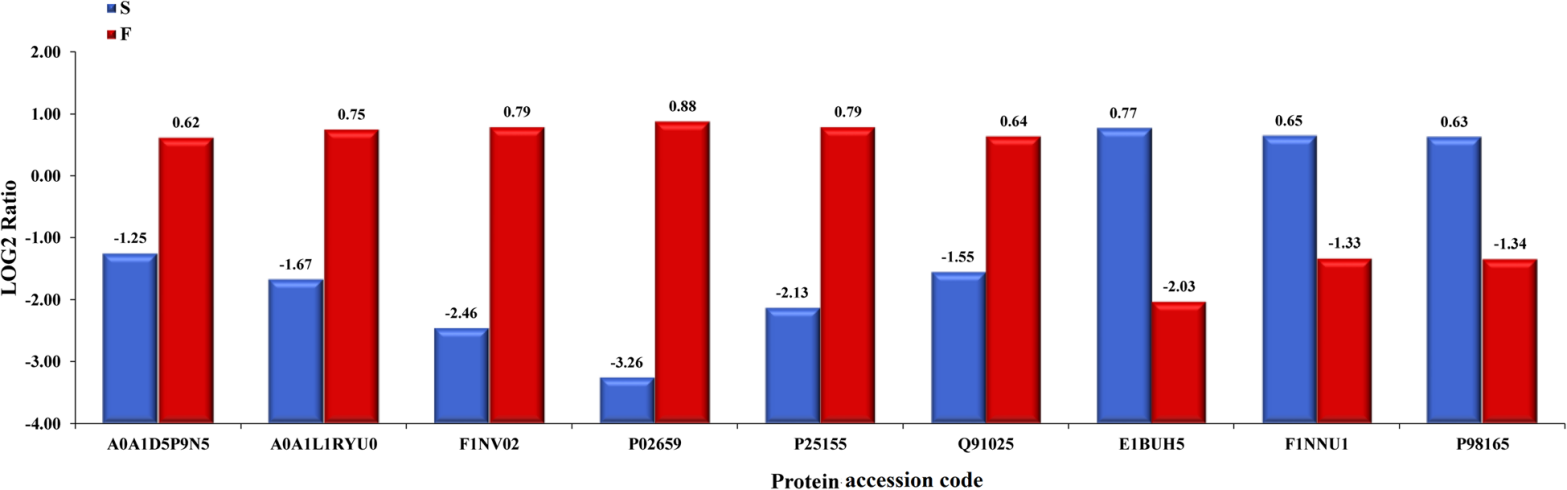
The histogram of the nine significantly abundant proteins in F6 follicles (F) vs SY follicles (S) according to PRM (*P* < 0.05)

Nine DEPs were randomly selected for parallel reaction monitoring (PRM) analysis to verify the accuracy of the proteome analysis by LC-MS/MS, including apovitellenin-1 (APO1), apolipoprotein B (APOB), prostate stem cell antigen (PSCA), coagulation factor X (F10), vitellogenin-1 (VTG1) and vitellogenin-3 (VTG3); all of these proteins were significantly increased in F6 follicles, and zona pellucida sperm-binding protein 2 (ZP2), zona pellucida sperm-binding protein 3 (ZP3) and very low-density lipoprotein receptor (VLDLR) were significantly decreased in F6 follicles (Table 4). The PRM results (Fig. 4) showed that the relative abundances of the peptides from the nine selected individual proteins were consistent with the proteome data.

**Table 4:** Nine proteins selected for the parallel reaction monitoring analysis of the chicken follicle proteome data

Protein accession code	Protein description	Protein name	Molecular mass (kDa)	F/S ratio	F/S ***P*** value	Regulation
A0A1D5P9N5	Vitellogenin-1	VTG1	209.88	3.858272907	0.029564918	Up
A0A1L1RYU0	Prostate stem cell antigen	PSCA	13.282	6.297115385	0.007551582	Up
F1NV02	Apolipoprotein B	APOB	523.35	6.922300706	0.026918903	Up
P02659	Apovitellenin-1	APOV1	11.966	10.52430044	0.018057763	Up
P25155	Coagulation factor X	F10	53.141	6.062727273	0.021845706	Up
Q91025	Vitellogenin-3	VTG3	38.15	4.500719424	0.03965785	Up
E1BUH5	Zona pellucida sperm-binding protein 3	ZP3	51.115	0.519704433	0.007108315	Down
F1NNU1	Zona pellucida sperm-binding protein 2	ZP2	77.033	0.534041942	0.040004135	Down
P98165	Very low-density lipoprotein receptor	VLDLR	94.904	0.551820728	0.019373296	Down

### Transcriptome and proteome association analysis

**Fig. 5:**
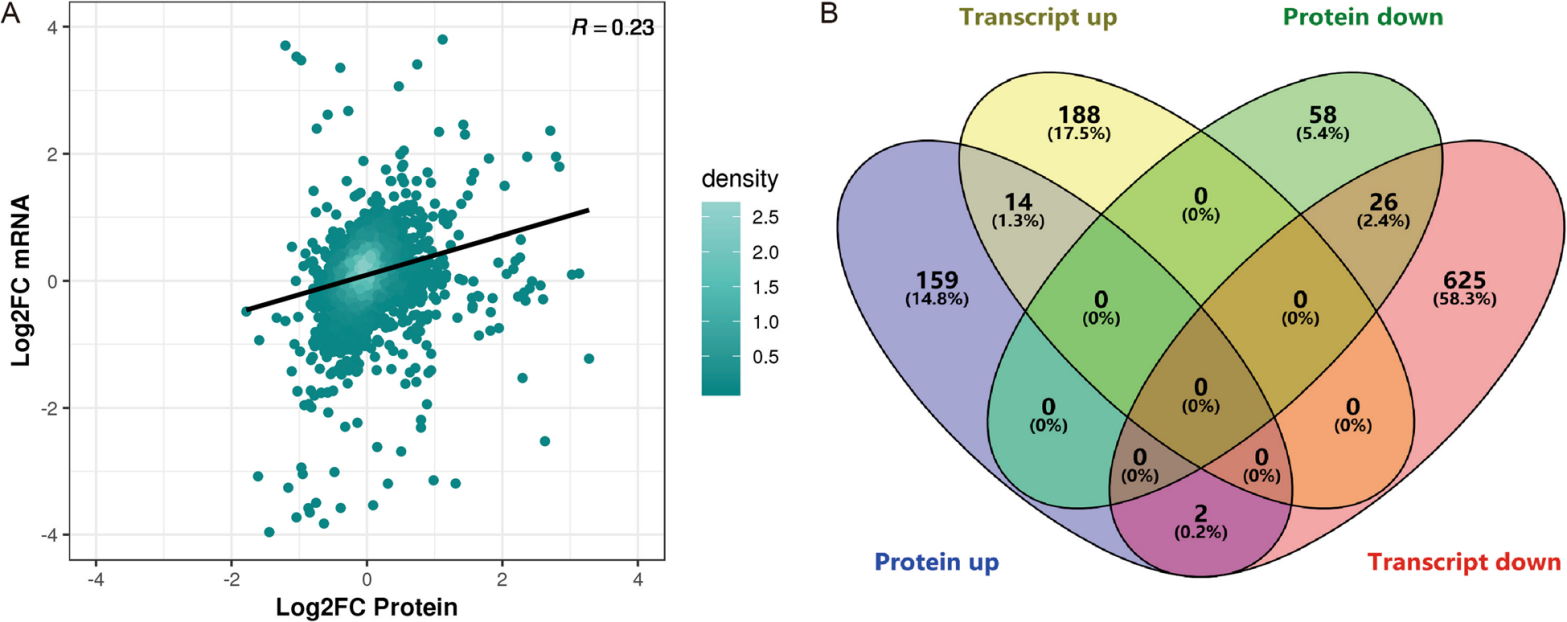
Association analysis (**a**) and Venn diagram (**b**) of differentially expressed genes/proteins from the TMT and DEG analyses between F6 and SY follicles in laying hens

**Fig. 6:**
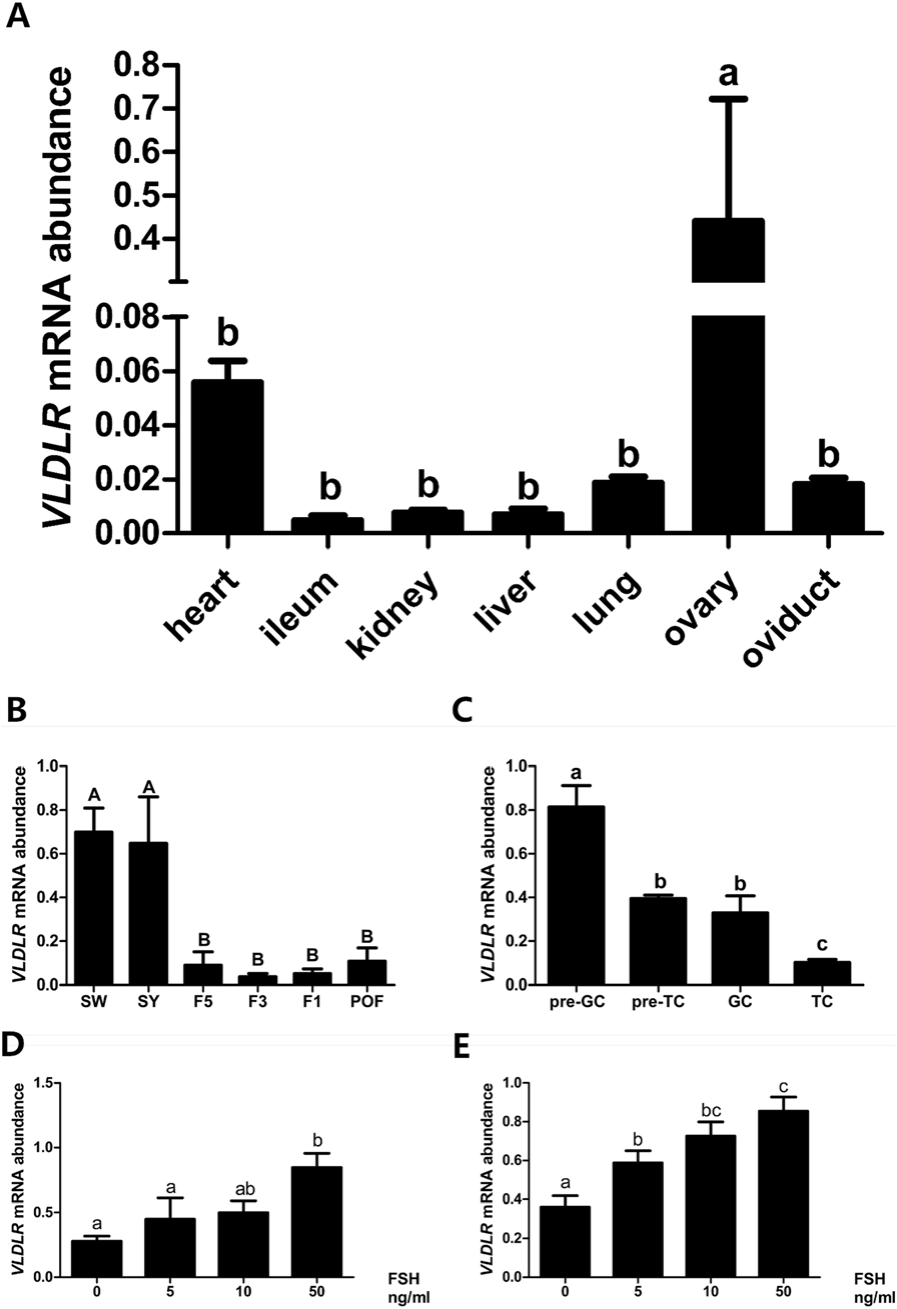
Dynamics of the expression of chicken *VLDLR* mRNA and the effect of follicle-stimulating hormone (FSH) treatment on the *VLDLR* mRNA levels in the granulosa cells (GCs) of chicken ovarian follicles. **a** Expression of *VLDLR* mRNA in different chicken tissues. **b** Expression levels of *VLDLR* in 1–2 mm follicles, 6–8 mm follicles (small yellow follicles), the fifth largest follicles (F5), the third largest follicles (F3), the largest follicles (F1), and the new postovulatory follicles (POFs). **c** Expression of chicken *VLDLR* in the granulosa cells (pre-GCs) and theca cells (pre-TCs) of prehierarchical follicles and the GCs and TCs of hierarchical follicles. **d** Effect of FSH on *VLDLR* in the GCs of prehierarchical follicles. **e** Effect of FSH on *VLDLR* in the GCs of hierarchical follicles. All data are presented as the mean ± SEM. (^abc^*P* < 0.05; ^ABC^*P* < 0.01)

The association analysis of the proteomic and transcriptomic data of the F6 and SY follicles revealed a weak relationship between protein and mRNA expression with a Pearson’s correlation coefficient of 0.23 (Fig. 5a). The number of items for which “Transcript up and Protein up” and “Transcript down and Protein down” was 14 (1.3%) and 26 (2.4%), respectively (Fig. 5b). To further understand the relationship between transcripts and proteins, we compared the intersection between DEGs and DEPs (Fig. 6). Most genes were significantly expressed at the mRNA level but not at the protein level. At both the protein and mRNA levels, 14 and 26 genes were revealed as significantly up- and downregulated in SY follicles compared with F6 follicles, respectively. In addition, the expression of two genes were inconsistent in terms of changes in the mRNA levels and protein levels. Table 5 shows the specific regulation information for several genes at the mRNA and protein levels. The specific comparative analysis results are shown in Table S4.

**Table 5:** Differentially expressed genes at both the mRNA and protein levels between SY and F6 follicles in chicken

Gene	Transcript			Protein		
	Log_2_ FoldChange	FDR	Regulation type	F/S ratio	*P*-value	Regulation type
*VLDLR*	−1.93821454837682	0.000000000251	Down	0.552	0.0194	Down
*NGFR*	−1.99046510645846	1.07E-12	Down	0.566	0.0264	Down
*WIF1*	−3.079759991	2.56E-20	Down	0.328	0.0128	Down
*KPNA7*	−5.083462853	1.76E-18	Down	0.581	0.0362	Down
*ACTC1*	3.404736805	0.000513608	UP	1.673	0.00158	UP
*LCAT*	2.363270714	2.85E-19	UP	6.531	0.0267	UP
*ZPD*	2.302937808	0.007012137	UP	2.727	0.0061	UP
*ACTA1*	1.543125393	0.000000000407	UP	1.923	0.00953	UP
*AGT*	−3.192564345	0.003115348	Down	2.478	0.00904	UP
*A2 ML1*	−1.15166374	0.000215557	Down	1.929	0.0304	UP

### Dynamics and regulation of VLDLR mRNA by FSH

Both transcriptomic and proteomic analyses indicated that VLDLR expression was significantly downregulated in F6 follicles compared with SY follicles; therefore, we further analyzed the expression of *VLDLR* mRNA in chicken tissues and found that it was predominantly expressed in the ovary (Fig. 6a), and VLDLR expression in the prehierarchical follicles was significantly higher than that in the hierarchical follicles (*P* < 0.01) (Fig. 6b). In both the hierarchical and prehierarchical follicles, the *VLDLR* mRNA expression was significantly higher in the granulosa cells (GCs) than in the thecal cells (TCs) (*P* < 0.05) (Fig. 6c). Follicle-stimulating hormone (FSH) treatment stimulated the expression of *VLDLR* in the GCs, in prehierarchical follicles, the effect was not significant at concentrations of ≤10 ng/ml (Fig. 6d). However, FSH treatment stimulated the expression of *VLDLR* in the GCs of hierarchical follicles in a dose-dependent manner (*P* < 0.01) (Fig. 6e).

## Discussion

Follicle selection is an important stage of follicle development that affects many egg production traits in the poultry industry. The mechanism of follicle selection in chickens is becoming a hot topic of research in poultry reproduction biology. Previous studies revealed the effect of Wnt4 [9], bone morphogenetic protein 4 [11], BMP15 [12], AMH [24], vasoactive intestinal peptide [25] and parathyroid hormone-like hormone [26] on chicken follicle selection and the changes in the RNA N6-methyladenosine methylation profile [27] during chicken follicle selection; however, high-throughput screening of functional genes at the protein level involved in chicken follicle selection is lacking. Therefore, in this study, by combined analysis of changes in the transcriptomic and proteomic profiles of follicles *prior to* and *post* selection in chickens, we revealed several DEGs and DEPs, including VLDLR, that may play important roles in chicken follicle selection.

At both the mRNA and protein levels, the expression of VLDLR, NGFR and WIF1 were significantly downregulated in chicken F6 follicles compared with that in SY follicles. During chicken follicle selection, VLDLR plays a pivotal role in the absorption of vitelin by oocytes, and without VLDLR, oocytes are unable to enter the rapid growth stage of follicle development [28]. During the development of the small white follicle, VLDLR migrates to the follicular wall, enabling the endocytosis of vitellogenin into the yolk, followed by follicular differentiation [7]. Studies have revealed that the expressed variant of *VLDLR* in chicken granulosa cells differs from the variant expressed in the oocyte, which contains an O-linked sugar domain (VLDLR 1) [29, 30], and the reduced level of VLDLR in granulosa cells is suggested to allow more VLDLR to reach the oocytes by passing through intercellular gaps rather than via receptor-mediated endocytosis into granulosa cells [31]. In this study, we provided further evidence that after follicle selection, the expression of *VLDLR* was significantly decreased. Moreover, we found that *VLDLR* is mainly expressed in chicken ovaries, SW and SY follicles and the GCs of prehierarchical follicles, and its expression was stimulated by FSH in the GCs, especially in those of hierarchical follicles. These data collectively suggest that, after follicle selection, the decreased expression of VLDLR in GCs might allow more VLDLR 1 to be expressed on the oocyte membrane, thus promoting the rapid growth of hierarchical follicles. Similarly, in geese, the expression of *VLDLR* mRNA is decreased concomitant with an increase in the follicular diameter [32]. For NGFR, in human mural and cumulus granulosa cells, the nerve growth factor receptor tropomyosin-related kinase A (TrkA) mRNA level was strongly correlated with the number of oocytes retrieved, and the number of oocytes retrieved was greater among women with a low p75(NTR)/TrkA ratio [33]. The role of WIF1 has not been reported in ovarian function or follicle growth. The function of NGFR and WIF1 in chicken follicle selection requires further investigation.

Hundreds of DEGs were identified by transcriptome comparison between SY and F6 follicles during chicken follicle selection. Among these DEGs, the expression of AMH and BMP15 was greatly decreased in F6 follicles compared with that in SY follicles. Similar dynamics for AMH and BMP15 expression were previously reported; AMH is mainly expressed in granulosa cells of 1–5 mm follicles in the early stage of follicular development [1] and is decreased in follicles after selection [24], while BMP15 may promote follicle selection and affect granulosa cell proliferation and steroidogenesis in hens [12].

Among the 259 DEPs identified by global proteome analysis of chicken SY and F6 follicles, the following proteins may play certain roles in chicken follicle selection. APO1 is one of five apoproteins that forms low-density lipoprotein (LDL) and is expressed in the egg yolk and vitelline membrane [34, 35]. APOB is the major apolipoprotein in very low-density lipoprotein (VLDL) and LDL and is a ligand for LDL receptors. The increased expression of APOV1 and APOB in F6 follicles is consistent with higher yolk incorporation after follicle selection. Vitellogenins are yolk precursor proteins produced by the liver and are essential for the growth of chicken ovarian follicles, and they circulate in the bloodstream until a follicle enters the stage of vitellogenesis, which triggers endocytosis of vitellogenins and transport into the yolk [6]. Vitellogenins 1, 2, and 3 were found in chicken egg yolk plasma and granules [35]. The increased expression of VTG1 and VTG3 in F6 follicles found in this study suggests that yolk deposition is active in F6 follicles. The zona pellucida (ZP) is a specialized extracellular matrix that surrounds the oocyte and early embryo and is composed of three or four glycoproteins, including zona pellucida sperm-binding proteins 1–4 (ZP1–4), with various functions in oogenesis and fertilization [36]. ZP3 has been shown to promote oocyte maturation in pigs [37]. In this study, we found that the expression levels of ZP2 and ZP3 were decreased to approximately half of the level found in F6 follicles; the mechanism of this process in follicle selection requires further study.

In this study, by association analysis between the transcriptome and the proteome, we found that the expression changes of most genes are inconsistent at the mRNA and protein levels, which is in line with the results of other studies showing that most genes can exhibit differential expression at the transcript and protein levels [38, 39], and this suggests that it is necessary to analyze the functions of genes at both levels.

## Conclusions

By comparing the transcriptomes and proteomes of SY follicles and F6 follicles in laying hens, this study revealed 855 DEGs and 259 DEPs. The possible functional significance of several DEGs and DEPs, including VLDLR, was further discussed. These data may contribute to the identification of the functional genes and proteins involved in chicken follicular development and selection.

## Methods

### Tissue collection

Fifteen randomly sampled Hy-line brown hens from the same batch, which had been laying regularly for at least 1 month (28 weeks old, with a mean body weight of 2.1 ± 0.12 kg) and were housed under standard conditions with free access to food and water, were divided into three biological groups, each containing five hens. Vaccination was performed according to the recommendations from Hy-line International. The experimental hens were killed by cervical dislocation after approximately 10 h after laying, the time of which was individually recorded. From each hen, small yellow follicles (6–8 mm in diameter, SY) and smallest hierarchical follicle (12–15 mm in diameter, F6) were separately collected, and the egg yolk was carefully squeezed out with tweezers, washed with phosphate-buffered saline (Thermo Fischer Scientific, MA, USA), immediately frozen in liquid nitrogen and used for transcriptomic and proteomic analyses. The Institutional Animal Care and Use Ethics Committee of Shandong Agricultural University reviewed and approved all procedures described in this study. This study was performed according to the Guidelines for Experimental Animals of the Ministry of Science and Technology of China. Three biological replicates were prepared for the transcriptomic and proteomic analyses of total RNA and proteins.

### Transcriptome analysis

Total RNA was isolated from follicles and follicular granulosa and thecal cells with TRIzol reagent (Invitrogen, CA, USA). The purity and integrity of total RNA were checked using the NanoPhotometer® spectrophotometer (IMPLEN, CA, USA) and the RNA Nano 6000 Assay Kit of the Bioanalyzer 2100 system (Agilent Technologies, CA, USA), respectively. Sequencing libraries were generated using NEBNext® UltraTM RNA Library Prep Kit for Illumina® (NEB, USA) following manufacturer’s recommendations and index codes were added to attribute sequences to each sample. The library preparations were sequenced on an Illumina Novaseq platform. Clean data (clean reads) were obtained by removing reads containing adapter, reads containing ploy-N and low quality reads from raw data. The index of the reference genome was built and the paired-end clean reads were aligned to the *Gallus gallus* genome (ftp://ftp.ncbi.nlm.nih.gov/genomes/all/GCF/000/002/315/GCF_000002315.6_GRCg6a/) using Hisat2 v2.0.5 [40]. Gene expression level was quantified with featureCounts v1.5.0-p3 and FPKM [41].

Differentially expressed genes (DEGs) between SY and F6 follicles were identified according to the cretiera of adjusted *P*-value < 0.05 and a |log_2_FoldChange| > 1 according to DESeq2 [42]. Gene ontology (GO) enrichment analysis and Kyoto Encyclopedia of Genes and Genomes database (KEGG) pathway enrichment analysis of the DEGs were implemented by the cluster Profiler R package, and genes with *P*-values less than 0.05 were considered significantly enriched as differentially expressed [43]. The transcriptome data have been deposited with the NCBI Sequence Read Archive (SRA, https://www.ncbi.nlm.nih.gov/sra/docs/) under accession number SRP236909.

### Sample processing and liquid chromatography coupled with tandem mass spectrometry (LC-MS/MS)

The proteins in the tissues were extracted with lysis buffer containing 8 M urea (Sigma Aldrich, MO, USA) and 1% protease inhibitor cocktail (Merck Millipore, MA, USA), their concentration was determined with a BCA kit (Beyotime, Shanghai, China) according to the manufacturer’s instructions. Protein enzymolysis was performed using trypsin (Promega, WI, USA). The peptides were desalted on a Strata X C18 SPE column (Phenomenex, CA, USA) and vacuum-dried after trypsin digestion, reconstituted in 0.5 M TEAB and processed according to the manufacturer’s protocol for the TMT kit (Thermo Fischer Scientific, MA, USA). The tryptic peptides were fractionated by high-pH reverse-phase HPLC using an Agilent 300 Extend C 18 column (5 μm particle size, 4.6 mm ID, 250 mm length), dissolved in 0.1% formic acid (Sigma Aldrich, MO, USA) (solvent A) and separated using EASY-nLC 1000 ultra-high performance liquid phase system. Finally, peptides that were separated were exposed to an NSI source followed by tandem mass spectrometry (MS/MS) with a Q Exactive™ Plus spectrometer (Thermo Fischer Scientific, MA, USA) coupled online to the UPLC system.

The resulting MS/MS data were processed using the Maxquant search engine (v.1.5.2.8). The tandem mass spectra were searched against the *Gallus gallus* database (http://www.uniprot.org/proteomes/UP000000539, chicken proteome ID: UP000000539) concatenated with the reverse decoy database. The GO proteome was derived from the UniProt-GOA database (http://www.ebi.ac.uk/GOA/). The KEGG database was used to identify the enriched pathways. Proteins with a threshold of *P* < 0.05 and a fold change of > 1.5 or < 1/1.5 were identified as differentially expressed proteins (DEPs) between SY and F6 follicles. The MS proteomics data have been deposited in the ProteomeXchange Consortium (http://www.proteomexchange.org/) via the PRIDE partner repository with the dataset identifier PXD011470 (Username: reviewer39674@ebi.ac.uk, Password: AfwLJZRb).

### Parallel reaction monitoring

PRM used to validate protein abundance levels of APO1, APOB, PSCA, F10, VTG1 and VTG3 that were significantly increased in F6 follicles, and ZP2, ZP3 and VLDLR that were significantly decreased in F6 follicles obtained from TMT analysis. PRM analysis was carried out at the Jingjie PTM BioLab Co., Ltd. (Hangzhou, China) with experimental steps and parameter settings used according to the reference [44].

### Cell culture and cell treatment

Granulosa and thecal cells were prepared according to references [14, 45], respectively. Firstly, the yolk was carefully removed with ophthalmic forceps. Then, small yellow follicles were digested with 0.1% collagenase II (MP Biomedicals, Santa Ana, CA, USA) at 38 °C for 15 min to obtain the pre-GCs, for additional 30 min to obtain the pre-TCs. For hierarchical follicles, after the removal of yolk, granulosa cells were firstly separated from the theca externa cells with ophthalmic forceps, followed by treatent with 0.25% trypsin-EDTA (Gibco-BRL, NY, USA) at 38 °C for 15 min, while the thecal cells were obtained by digesting the theca externa and interna with 0.1% collagenase II at 38 °C for 30 min. After centrifugation, the cells were suspended in culture medium containing M199 (Gibco-BRL, NY, USA), 10% fetal bovine serum (Biological Industries, Kibbutz Beit Haemek, Israel) and 1% penicillin/streptomycin (Solarbio, Beijing, China) and subsequently seeded at a density of 1 × 10^5^ cells/well in 24-well culture plates and cultured at 38 °C in a water-saturated atmosphere of 95% air and 5% CO_2_. The number of viable cells was estimated using Trypan blue staining. After 24 h, cultured granulosa cells were treated with different concentrations (0, 5, 10, and 50 ng/ml) of recombinant FSH (R&D Systems, MN, USA). All the treated cells were collected after another 24 h for RNA extraction and qRT-PCR analysis.

### Real-time quantitative PCR

Total RNA was extracted from chicken ovarian follicles that were also used for proteome analysis using TRIzol reagent (InvivoGen, CA, USA). Synthesis of the cDNA was performed using a PrimeScript RT reagent kit with 1 μg of the RNA pretreated with gDNA Eraser (TaKaRa, Dalian, China) according to the manufacturer’s protocol. Real-time quantitative PCR of the mRNA expression level of *VLDLR, VLDLR1, WIF1, NGFR, AMH, BMP15, GDF6* and *MMP13* was performed using a SYBR Premix Ex Taq™ II kit (TaKaRa, Dalian, China) with primers listed in Table S5 on a Light Cycler 480 real-time PCR system (Roche, Basel, Switzerland) as follows: 95 °C for 30 s, followed by 40 cycles of denaturation at 95 °C for 10 s and annealing and extension at 58 °C for 20 s. The melting curves were obtained, and quantitative analysis of the data was performed using the 2^−ΔΔCT^ relative quantification method [46]. Quantification was performed by standardizing the reaction results versus those of *β-actin*.

### Statistical analysis

Differences in mRNA expression between follicles and follicular cells were evaluated by one-way ANOVA followed by Duncan’s multiple range test (*P* < 0.05) using the General Linear Model procedure in SAS (version 9.2). For one experiment, each treatment was repeated four times, and at least three independent experiments were performed. All data are presented as the mean ± SEM (*n* = 4). The GO and KEGG analyses were performed by using Fisher’s t-test. *P* < 0.05 was considered to indicate a significant difference.

## Supplementary information

**Additional file 1: Table S1.** DEGs. (XLS 315 kb)

**Additional file 2: Table S2**. Seven DEGs selected for qRT-PCR validation in chicken follicles. (DOCX 13kb)

**Additional file 3: Table S3.** DEPs between F and S. (XLSX 171kb)

**Additional file 4: Table S4.** Protein and Transcript quantiation combine. (XLS 35 kb)

**Additional file 5: Table S5.** The primers used in the experiments. (DOCX 20kb)

## Data Availability

The reference genome (ftp://ftp.ncbi.nlm.nih.gov/genomes/all/GCF/000/002/315/GCF_000002315.6_GRCg6a/) and proteome (http://www.uniprot.org/proteomes/ UP000000539, chicken proteome ID: UP000000539) database of the *Gallus gallus* were used in this study. The transcriptome data have been deposited with the NCBI Sequence Read Archive (SRA, https://www.ncbi.nlm.nih.gov/sra/docs/) under accession number SRP236909. The MS proteomics data have been deposited in the ProteomeXchange Consortium (http://www.proteomexchange.org/) via the PRIDE partner repository with the dataset identifier PXD011470 (Username: reviewer39674@ebi.ac.uk, Password: AfwLJZRb).

## Abbreviations

BP: Biological process
CC: Cellular component
DEGs: Differentially expressed genes
DEPs: Differentially expressed proteins
GO: Gene ontology
KEGG: Kyoto Encyclopedia of Genes and Genomes
GCs: granulosa cells
TCs: thecal cells
