# Supplementary material for: Transcriptomic and proteomic analyses of ovarian follicles reveal the role of VLDLR in chicken follicle selection
Source: BMC Genomics. 2020 Jul 16;21:486. doi: 10.1186/s12864-020-06855-w (PMC7367319; doi:10.1186/s12864-020-06855-w)
Supplement: Supplementary file 2 — Additional file 2: Table S2. Seven DEGs selected for qRT-PCR validation in chicken follicles. (DOCX 13kb) [file 12864_2020_6855_MOESM2_ESM.docx]

**Table S2**. Seven DEGs selected for qRT-PCR validation in chicken follicles

| Gene ID | Gene name | Gene description | Log2Foldchange | padj | Regulation |
| --- | --- | --- | --- | --- | --- |
| 396154 | VLDLR1 | very low density lipoprotein receptor transcript variant X1 | -1.93821454837682 | 2.52E-10 | Down |
| 425805 | NGFR | nerve growth factor receptor | -1.99046510645846 | 1.07E-12 | Down |
| 417831 | WIF1 | WNT inhibitory factor 1 | -3.07976009002215 | 2.58E-20 | Down |
| 395887 | AMH | anti-Mullerian hormone | -3.49764002338442 | 8.03E-28 | Down |
| 428697 | BMP15 | bone morphogenetic protein 15 | -2.82700723287993 | 8.58E-07 | Down |
| 10705276 | GDF6 | growth differentiation factor 6 | 2.17362412753088 | 0.004248664 | Up |
| 395683 | MMP13 | matrix metallopeptidase 13 | 2.05982002438789 | 5.10E-12 | Up |
