# Supplementary material for: Transcriptomic and proteomic analyses of ovarian follicles reveal the role of VLDLR in chicken follicle selection
Source: BMC Genomics. 2020 Jul 16;21:486. doi: 10.1186/s12864-020-06855-w (PMC7367319; doi:10.1186/s12864-020-06855-w)
Supplement: Supplementary file 5 — Additional file 5: Table S5. The primers used in the experiments. (DOCX 20kb) [file 12864_2020_6855_MOESM5_ESM.docx]

Table S5 The primers used in the experiments

| Gene Symbol | Accession Number (GenBank) | Primer Sequence | Product Size |
| --- | --- | --- | --- |
| *VLDLR1* | XM_015280076.2 | Forward: 5’TGCCTGCTCCTCAGATAAA  Reverse: 5’GTAAGCCACAGTTGTTCCAGT | 122 bp |
| *VLDLR* | NM_205229.1 | Forward: 5’TGTGCCTGCCTGCTCCTCAG  Reverse: 5’ CCTCCACATCTCAGACCGTCCTC | 100 bp |
| *NGFR* | NM_001146133.1 | Forward: 5’ GAACGAGGTGATGGTGAA  Reverse: 5’ ATGACGGTGGTGACAATG | 183 bp |
| *WIF1* | NM_001199607.2 | Forward: 5’ TCAGTGGTTCAGGTTGGGTT  Reverse: 5’ TCCTGGACACTCCGCTTG | 159 bp |
| *AMH* | NM_205030.1 | Forward: 5’CCCCTCTGTCCCTCATGGA  Reverse: 5’CGTCATCCTGGTGAAACACTTC | 70 bp |
| *BMP15* | NM_001006589.2 | Forward: 5’ACGGCAGCATCCTCTACAAG  Reverse: 5’CACCCACCAAGCATCAAA | 114 bp |
| *GDF6* | XM_015282935.2 | Forward: 5’CGCTCTCTTTCGCATTCCT  Reverse: 5’TCACGCCTCGCTCTTATCA | 181 bp |
| *MMP13* | NM_001293090.1 | Forward: 5’TTTGGATTAGAGGTGACGG  Reverse: 5’CCACTTCGTATTCTGGTGA | 253 bp |
| *β-actin* | K02173.1 | Forward: 5’ TGGATGATGATATTGCTGC  Reverse: 5’ ATCTTCTCCATATCATCCC | 253 bp |
